# Mapping COVID-19 Artificial Intelligence (AI) Research in Medical Imaging: A Bibliometric Analysis of Datasets, Trends, and Clinical Challenges

**DOI:** 10.7759/cureus.111190

**Published:** 2026-06-20

**Authors:** Patrice Menoudji Djetoyom, Alladoumbaye Ngueilbaye, Adam Abakar Hamid, Atitso Akofala

**Affiliations:** 1 College of Bioinformatics Science and Technology, Harbin Medical University, Harbin, CHN; 2 School of Artificial Intelligence, Shenzhen University, Shenzhen, CHN; 3 College of Computer Science and Artificial Intelligence, Wenzhou University, Wenzhou, CHN; 4 College of Biochemistry and Molecular Biology, Harbin Medical University, Harbin, CHN

**Keywords:** bibliometric analysis, computed tomography, convolutional neural network, covid-19, vosviewer, x-ray

## Abstract

The rapid adoption of artificial intelligence (AI) for COVID-19 pandemic diagnosis has exposed critical gaps in medical imaging datasets. This Preferred Reporting Items for Systematic Reviews and Meta-Analyses (PRISMA)-compliant bibliometric review of 450 PubMed studies (2020-2024) reveals that only 21.5% of the datasets remain clinically validated, while 55% are unavailable or repurposed from non-COVID-19 sources. We identified persistent issues, such as resolution heterogeneity and radiologist annotation scarcity, that undermine model reliability. Numerous convolutional neural network (CNN) architectures have been developed to enable fast and accurate automated diagnosis of COVID-19 using computed tomography (CT) or X-ray imaging. However, due to the urgency of the pandemic and the rapid demand for solutions, existing computer-aided diagnostic (CAD) systems face several critical limitations, such as imbalanced datasets, insufficient bias assessment in model training, and inconsistent quality control in image acquisition and preprocessing. In this bibliometric analysis, we provide an analysis of PubMed articles on COVID-19 imaging published between January 1, 2020, and November 1, 2024. The research included 1261 publications. VOSviewer was used to generate a visual map of the keyword networks and authors. The journal with the most publications was Elsevier, and the most used dataset was the COVID-19 Radiography Database from Kaggle.

## Introduction and background

COVID-19 has been one of the most devastating scourges in human history. The health systems in all countries around the world have been strained to contain the disease, but despite this, the pandemic has claimed more than 7.01 million lives worldwide to date [1]. There was an urgent need to create trustworthy tools for the diagnosis and prognosis of the disease. A fast, accurate, and non-invasive method of COVID-19 diagnosis was the ideal solution to increase testing capacity and minimize further community transmission. Although most patients with COVID-19 infection did not develop pneumonia, early identification of COVID-19-induced pneumonia cases is essential for monitoring patients [2]. As a result, medical imaging has become increasingly in demand, and the guiding roles of imaging in the diagnosis and evaluation of pneumonia rehabilitation have been increasingly recognized. Medical imaging can be applied to all stages of COVID-19 with respect to diagnosis, treatment guidance, and prognosis prediction [3-6]. In pursuit of this objective, imaging modalities such as planar X-ray, computed tomography (CT), and sometimes ultrasound were employed. Publications related to the detection of COVID-19 through medical imaging were increasing rapidly [7]. However, the rapid surge in publications can be overwhelming for researchers, making it difficult to gain a comprehensive understanding of the field's major developments. Consequently, it is essential to perform a bibliometric analysis of the COVID-19 imaging literature produced during this period [8-10]. Bibliometric analysis applies mathematical and statistical techniques to quantitatively examine research publications on a particular subject. This method also helps evaluate study quality, track the progression of research trends, and forecast possible future research directions. Analyzing COVID-19 literature through bibliometric methods from various angles helps uncover key research priorities during the pandemic, highlights emerging viewpoints and future directions in the field, and aims to offer valuable guidance for both academic researchers and policymakers working together [11-13]. However, one of the key problems in developing artificial intelligence (AI) models for COVID-19 detection was the limited size of COVID-19 medical datasets. Most of the publicly available datasets have been created by various research organizations. Nevertheless, the availability of high-quality COVID-19 CT scans and X-ray images that have been properly diagnosed is still very limited and imbalanced.

This comprehensive review aims to systematically review published studies from PubMed that applied deep learning approaches for the diagnosis and prognosis of COVID-19 based on CT and X-ray images from January 1, 2020, to November 1, 2024. We analyze publicly available CT and X-ray imaging datasets for COVID-19 cases. Many of the datasets used in some articles are no longer available. Therefore, we have compiled an updated list of datasets while highlighting the imbalanced number of COVID-19 images compared to normal and other pneumonia images in the literature. VOSviewer was used to analyze 1261 publications and generate knowledge maps. Our main contributions to the three unsolved questions are as follows: (a) Data availability and accessibility (What proportion of COVID-19 imaging datasets referenced in PubMed-indexed studies remain publicly accessible, and what are their key attributes (e.g., modality, sample size, patient demographics)?); (b) bias and heterogeneity (To what extent do current datasets suffer from class imbalance, variability in imaging protocols (e.g., resolution, acquisition parameters), and inconsistent annotation standards?); and (c) methodological evolution (How have dataset adoption trends and deep learning approaches (e.g., architectures, preprocessing techniques) for the diagnosis of COVID-19 evolved between 2020 and 2024?).

We present a Preferred Reporting Items for Systematic Reviews and Meta-Analyses (PRISMA)-compliant bibliometric analysis of 450 PubMed studies to (a) quantify dataset attrition rates, (b) analyze imbalances in COVID-19 versus non-COVID-19 images, and (c) provide a curated list of validated datasets for future research. Our findings reveal that only 21.5% of datasets meet clinical reliability standards, while 55% are either unavailable or repurposed from non-COVID-19 sources. These insights aim to guide radiologists in selecting datasets and motivate AI researchers to adopt standardized reporting practices.

The role of AI in diagnostic imaging has received significant attention in recent years, with many studies showing its potential to enhance the precision and efficiency of medical image interpretation. Khalifa and Albadawy [14] emphasize how AI can improve the analysis of X-rays, magnetic resonance images (MRIs), and CT scans, underscoring its growing importance in clinical practice. The need for AI adoption became particularly evident during the COVID-19 pandemic, when AI-driven solutions were rapidly implemented to help detect and manage the disease through the analysis of chest radiographs and CT scans [15]. This period saw a surge in research activity, which emphasized the application of AI in diagnostic precision, risk assessment, and evaluation of treatment responses for COVID-19. The proliferation of healthcare data, driven by advancements in digital technologies and big data analysis, has played a crucial role in these developments [16]. However, despite the progress made, there are still challenges in integrating AI into everyday clinical workflows, necessitating further investigation to overcome barriers to its widespread use.

Recent work highlights the crucial role AI models are expected to play in prioritizing cases across various medical conditions, enabling timely diagnoses and interventions [17]. AI algorithms have demonstrated impressive proficiency in analyzing medical images, such as spotting early-stage cancers, fractures, and infections that might be overlooked by human observers [18]. Several case studies have proposed numerous deep learning approaches to enhance performance by combining various classification techniques and machine learning methods. A study proposed transfer learning and parameter optimization to simultaneously classify X-ray and CT images under a hierarchical architecture [19], while another study proposed transferring an ensemble of 15 pre-trained models with data augmentation techniques [20]. Gupta et al. [21] claimed the effectiveness of convolutional neural network (CNN) models and proposed COVID-WideNet, a capsule network with 20 times fewer trainable parameters, making it computationally less expensive while preserving model performance. Feature optimization, channel boosting, and recurrent units such as recurrent neural network (RNN) or long short-term memory (LSTM) have also demonstrated enhanced performance in studies using the Advanced Squirrel Search Optimization Algorithm [22], Channel-Boosted Split-Transform-Merge Region and Edge Network (CBSTM-RENet) [23], and Gated Recurrent Unit (GRU) models [24]. However, the most effective way to boost the performance of deep learning techniques remains the availability and quantity of high-quality datasets.

In the realm of cancer imaging, AI shows great potential to improve the way expert clinicians interpret images. Bi et al. [25] demonstrate that AI can track tumor volumes over time, derive biological information from radiographic phenotypes (image data), predict clinical outcomes, and assess how diseases and treatments affect nearby organs. These abilities enable AI to identify suspicious areas, measure disease severity, and monitor treatment responses, giving healthcare professionals actionable insights. The rapid growth of machine learning models in medical imaging, observed across both research institutions and industry, highlights the growing importance of AI in healthcare [26]. The intersection of AI and medical imaging has led to major improvements in clinical practice, with AI algorithms playing a greater role in analyzing complex images and supporting clinical decision-making [27]. The ability of AI to analyze medical images with exceptional efficiency and accuracy brings computerized systems closer to matching the diagnostic capabilities of human clinicians [28].

The rapid advancement of deep learning methods has greatly increased the availability of large-scale, labeled open-access datasets. Studies have shown that the dataset sample size plays an important role in model performance. In general, models trained on small-sized datasets often demonstrate weaker generalization, although some models still achieve high accuracy despite limited data. For instance, Serte and Demirel [29] developed a ResNet-50 model for COVID-19 detection in 3D chest CT scans that outperformed other deep learning approaches, achieving 100% sensitivity, 98% specificity, and 96% area under the curve (AUC). Similarly, Sharifrazi et al. [30] proposed a hybrid CNN-support vector machine (SVM) framework for X-ray classification, achieving 100% sensitivity, 95.23% specificity, and 99.02% accuracy using 10-fold cross-validation.

Many studies have adopted ensemble learning approaches to enhance classification accuracy by combining multiple distinct CNN models to produce more reliable results. For example, one study achieved 99.31% accuracy using a stacked ensemble of ResNet50, ResNet101, VGG16, VGG19, Xception, MobileNetV1, MobileNetV2, DenseNet121, and DenseNet169. Another study employed a fuzzy membership-based ensemble of InceptionV3, DenseNet121, and VGG19 classifiers, further demonstrating the versatility of ensemble methods in this domain [31].

Various studies have applied additional preprocessing techniques prior to inputting data into deep CNN models, generally resulting in improved classification outcomes. Data augmentation was widely utilized, with most studies reporting high accuracy following its implementation. For instance, Zhao et al. [32] achieved 99% accuracy in a three-class classification task (non-COVID-19, COVID-19, pneumonia) using the ResNet-V2 architecture after applying augmentations such as random segmentation of scans into 480 × 480 pixels, random horizontal flipping, and normalization. Moreover, several studies incorporated lung segmentation techniques to isolate relevant regions, including the "snake" method employed by Saad et al. [33], which segments lung areas to remove noise and irrelevant background pixels.

The heterogeneity of a dataset significantly influences the classification performance of state-of-the-art deep learning models, as evidenced by the China Consortium of Chest CT Image Investigation (CC-CCII) dataset [34]. While a specific CNN model achieved the highest accuracy of 93% on this dataset [35], this performance remains suboptimal compared to deep learning models applied to other large datasets. To tackle these limitations, recent studies explored diverse deep learning approaches, combining several classification strategies and machine learning methods. For instance, Ghaderzadeh et al. [19] proposed transfer learning and parameter optimization to simultaneously classify X-ray and CT images in a hierarchical architecture, while Gifani et al. [20] suggested an ensemble of 15 pre-trained models enhanced with data augmentation. However, the best way to boost the performance of deep learning techniques is through the availability and quantity of quality datasets.

## Review

Review methodology

Research Design

**Figure 1:**
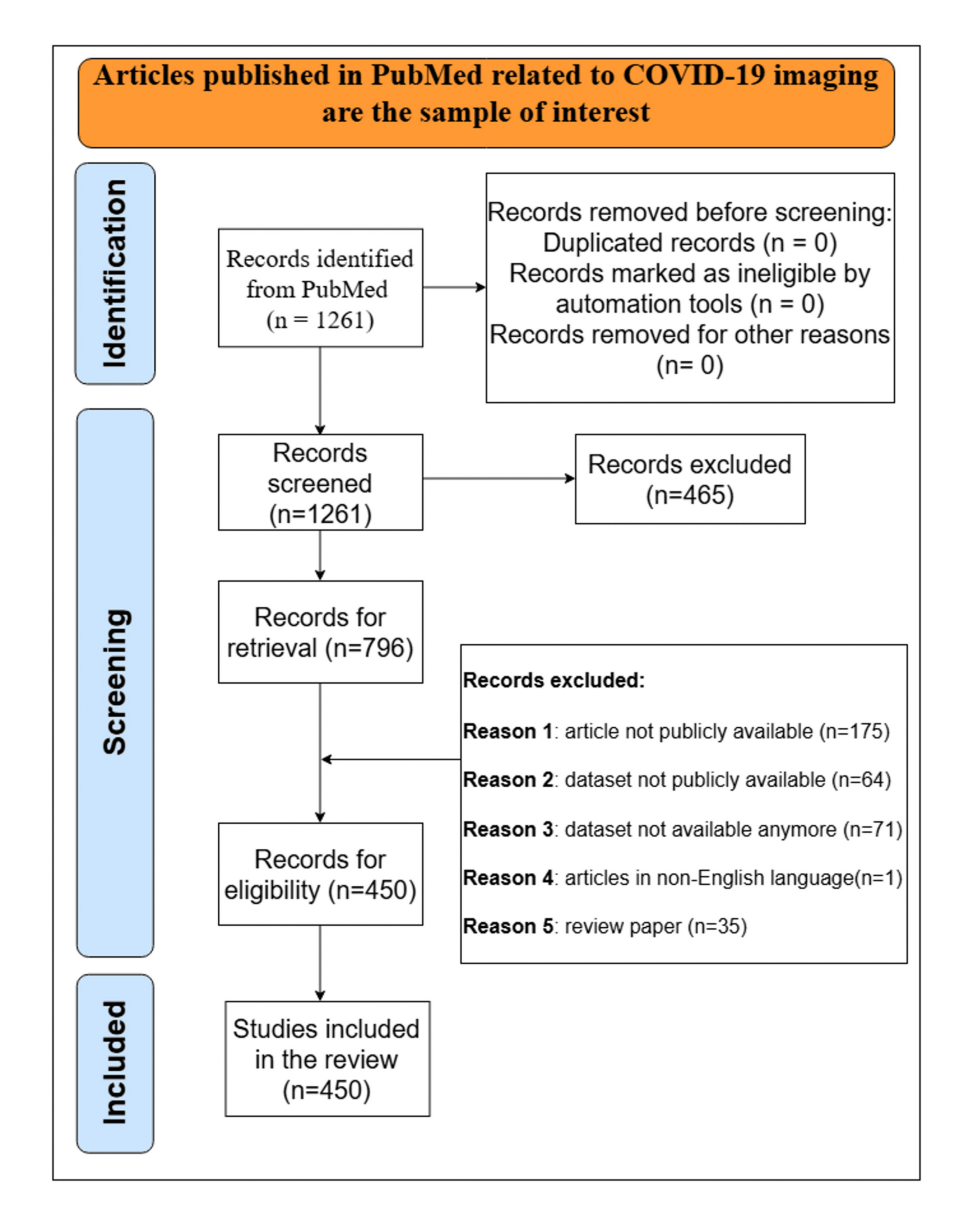
PRISMA flow diagram of the study PRISMA: Preferred Reporting Items for Systematic Reviews and Meta-Analyses

A scoping review of COVID-19-related medical imaging research was conducted following PRISMA guidelines as illustrated in Figure 1 [36].

Data Acquisition and Selection of Studies

A systematic literature search was conducted using the PubMed database. The sample of interest was the studies pertaining to COVID-19 and medical imaging. The search strategy combined the following terms: "COVID-19", "SARS-CoV-2", "2019 novel coronavirus", "SARS-CoV-2 Virus", "COVID-19 Virus", "coronavirus 2", "X-ray", "chest X-ray", "CT scan", "chest CT", "chest radiology", and "computed tomography". This review aims to achieve three main objectives: (1) to critically examine original studies as well as publicly available datasets related to the application of deep learning for COVID-19 detection; (2) to provide a detailed analysis of the imbalance between images of COVID-19 patients and those of healthy individuals or patients with other types of pneumonia; and (3) to formulate recommendations and highlight future perspectives and challenges based on the findings of this systematic review. The inclusion and exclusion criteria are outlined as follows: the included studies must have assessed the diagnosis of COVID-19 using deep learning algorithms in patients with pulmonary manifestations; review articles were excluded; articles in a non-English language were excluded; articles not publicly available, with datasets not available anymore, or that don't have publicly available datasets were excluded; studies that investigated diagnostic approaches for pulmonary manifestations of COVID-19 without employing deep learning techniques were likewise excluded from this review; and finally, studies focusing on non-radiological diagnostic approaches for pulmonary manifestations in COVID-19 patients were also excluded, even if deep learning techniques were employed.

The literature search identified 1261 references. Following the removal of duplicates, screening of titles and abstracts, and full-text review, a total of 450 publications were retained for analysis in the final version of this review. The selection of potential studies was conducted by a panel of four reviewers. One of the reviewers (PMD) defined the thematic framework and the "sample of interest", corresponding to studies published in PubMed focusing on COVID-19 and medical imaging, while another (AN) developed the search strategy and determined the terms used for database queries. After duplicates were removed, titles and abstracts were screened. Data extraction was performed by three reviewers (PMD, AAH, and AA). Any discrepancies were resolved by consensus.

Data Analysis

VOSviewer (version 1.6.20; https://www.vosviewer.com/download) was employed to conduct bibliometric analysis and network visualization. Using this tool, we generated knowledge maps highlighting keyword co-occurrence as well as author co-authorship.

Results

Keyword Co‑occurrence Analysis

**Figure 2:**
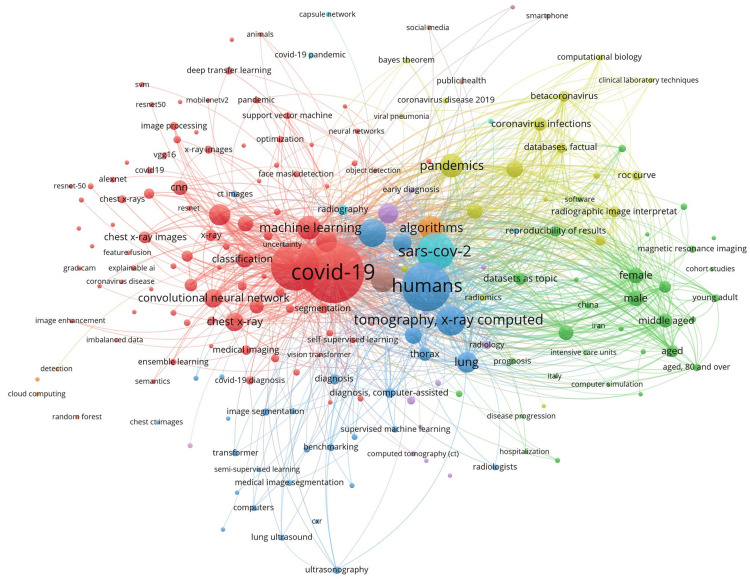
Map of the 201 leading keywords with co-occurrence frequency greater than 50 This figure was generated using VOSviewer (version 1.6.20; https://www.vosviewer.com/download).

Figure 2 presents a network visualization highlighting the 201 most frequent keywords, each with a co-occurrence frequency exceeding 50. In these knowledge maps, the nodes represent analytical entities such as keywords or authors, and the size of each node is proportional to the number of publications associated with it. The term "COVID-19" emerged as the most frequently used keyword. Table 1 lists the top 20 most prominent keywords characterized by strong burst strength, as identified in the scientific literature related to COVID-19 imaging research.

**Table 1:** Top 20 keywords NOO: number of occurrences; TLS: total link strength

Rank	Keyword	NOO	TLS
1	COVID-19	925	5060
2	Human	582	4213
3	Deep learning	576	3274
4	Sars-cov-2	348	2738
5	Computed tomography	219	1936
6	Neural network	193	1519
7	Algorithms	141	1204
8	Artificial intelligence	141	926
9	Machine learning	140	819
10	Pandemics	139	1214
11	Lung	110	981
12	X-rays	108	807
13	Pneumonia	102	784
14	COVID-19 testing	86	702
15	Image processing	75	594
16	Pneumonia, viral	71	815
17	Male	64	764
18	Female	64	753
19	Radiography, thoracic	64	629
20	Middle aged	48	626

Keyword analysis indicated that deep learning represents the third most frequently addressed topic in COVID-19-related medical imaging research. Deep learning networks are among the most advanced techniques for performing tasks such as image detection, segmentation, and classification in medical imaging [37-39]. The growth of these techniques has been largely driven by the increasing availability of large, well-annotated open-access datasets. Therefore, information on the datasets' origin, data types, sample size, image resolutions, and available links are crucial to achieve the highest and most reliable performance.

Analysis of Co‑authorship

**Figure 3:**
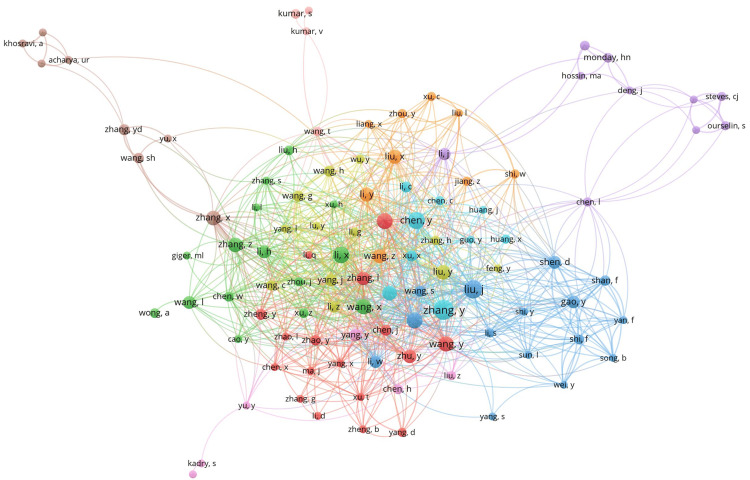
Authors' distribution and cooperation among them This figure was generated using VOSviewer (version 1.6.20; https://www.vosviewer.com/download).

Publications with a large number of co-authors were excluded from the analysis. A maximum threshold of 25 authors per document was established. Based on this, a total of 5553 authors were identified across all included publications. By setting a minimum of five publications per author, 129 authors met the inclusion threshold. Zhang Y. emerged as the most prolific contributor with 25 articles, followed by Liu J. with 19 and Chen Y. with 17. Table 2 lists the top 10 most productive authors, and Figure 3 illustrates the collaboration network among them.

**Table 2:** The top 10 most productive authors NOA: number of articles; TLS: total link strength

Rank	Author	NOA	TLS
1	Zhang Y.	25	49
2	Liu J.	19	58
3	Chen Y.	17	48
4	Li X.	16	38
5	Zhang J.	16	36
6	Wang Y.	14	43
7	Wang J.	13	39
8	Shen D.	11	55
9	Gao Y.	11	48
10	Shan F.	8	41

Analysis of Cited Journals

A total of 27 journals contributed to the publication of the 450 articles included in the final version of our review. Elsevier ranked first with 153 publications during the bibliometric analysis period, followed by Springer with 114 and Multidisciplinary Digital Publishing Institute (MDPI) with 51. Regarding co-cited journals, a total of 43,305 citations were recorded, with the most influential publication in the field cited 3605 times, accounting for 8.32% of all citations. Table 3 presents the top 10 journals in COVID-19-related imaging research.

**Table 3:** Top 10 journals in COVID-19-related imaging research NOP: number of publications; POC: percentage of citations; MDPI: Multidisciplinary Digital Publishing Institute

Rank	Journal	NOP	POC
1	Elsevier	153	34
2	Springer	114	25.3
3	MDPI	51	11.3
4	Scientific reports	28	6.2
5	Wiley	25	5.5
6	IEEE	20	4.4
7	Plos One	10	2.2
8	PeerJ	9	2
9	Sage Journals	9	2
10	Frontiers	7	1.5

Dataset Analysis

The availability of large, well-annotated open-access datasets has significantly supported the growth of deep learning methods. However, accessing publicly available datasets that contain a sufficient number of high-quality images for the effective use of deep learning methods remains a significant challenge. In addition, radiological images, such as chest X-rays and CT scans, are often acquired at different resolutions, complicating standardized processing.

These images were obtained from various sources, including intrinsic hospital datasets such as the Wuhan Pulmonary Hospital (China) [40], municipal hospitals in Moscow (Russia) [41], and São Paulo hospitals (Brazil) [42]. Some datasets were derived from open sources such as the CC-CCII dataset [34], MosMedData [41], COVID-CT [43], SARS-CoV-2 CT Scan [43], and ChestX-ray8 [44]. A portion of these datasets consists of aggregated collections of images from various open-source databases related to COVID-19 or other pulmonary diseases.

This heterogeneity raises concerns regarding data consistency, as radiological images, such as chest X-rays and CT scans, are often acquired at varying resolutions, including 512 × 512, 580 × 335, 333 × 308, 299 × 299, 1024 × 1024, 1766 × 1349, 224 × 224, and 1857 × 1317. In an effort to address the challenges and limitations associated with deep learning architectures in the context of COVID-19, Hasan et al. [45] trained models based on ResNet and Xception architectures to investigate potential issues such as overfitting, bias, and dataset-related limitations commonly encountered in research studies. They highlighted that the high accuracy reported in recent algorithms is often attributable to biases in experimental design and overfitting. Specifically, the use of cross-validation without an independent test set tends to lead to an overestimation of the model's capabilities. A major concern is that when data from different classes originate from distinct sources, the network may focus on dataset-specific features rather than relevant pathological characteristics [45].

This bibliometric analysis confirmed a similar trend among the articles published in PubMed during the review period. Among the 20 most-cited papers, the average number of datasets used was three, clearly indicating that researchers often had to merge datasets from various sources to obtain sufficient data representative of each pathology. Furthermore, the datasets exhibit significant imbalance, with thoracic disease images outnumbering COVID-19 images by approximately a factor of 10. This disproportion increases the risk of deep learning models overfitting to the majority class, thereby compromising generalization capability. Numerous studies have addressed the challenge of class-imbalanced data, spanning from conventional machine learning methods to more recent advancements in deep learning, with applications across disciplines such as management science and engineering [46].

To address data distribution imbalance, two basic approaches are commonly employed: random oversampling of minority classes and random undersampling of majority classes. A comprehensive analysis of the challenges associated with CNN applications has shown that oversampling generally yields more effective results, although undersampling may prove beneficial depending on the class ratio and the severity of the imbalance [47].

The issue of dataset imbalance was further assessed by aggregating all images from the databases included in this bibliometric analysis. A total of 342,141 images were identified, distributed as follows: 265,304 normal cases (77.54%), 63,523 pneumonia cases (18.57%), and 13,314 COVID-19 cases (3.89%). These findings reveal a pronounced class imbalance across the reviewed datasets, with substantial underrepresentation of COVID-19 cases. Such an imbalance may bias model training toward majority classes and potentially inflate performance metrics, particularly overall accuracy, while limiting generalizability and sensitivity for minority classes. Table 4 lists the 20 most-cited papers and the number of different datasets used in each study.

**Table 4:** The top 20 co-cited references NOC: number of citations; NOD: number of datasets

Rank	Title	Authors	NOC	NOD	Link
1	COVID-Net: a tailored deep convolutional neural network design for detection of COVID-19 cases from chest X-ray images	Wang et al.	3605	1	[48]
2	Covid-19: automatic detection from X-ray images utilizing transfer learning with convolutional neural networks	Apostolopoulos and Mpesiana	2626	5	[49]
3	Automatic detection of coronavirus disease (COVID-19) using X-ray images and deep convolutional neural networks	Narin et al.	2393	3	[50]
4	CoroNet: a deep neural network for detection and diagnosis of COVID-19 from chest x-ray images	Khan et al.	1478	2	[51]
5	Classification of COVID-19 in chest X-ray images using DeTraC deep convolutional neural network	Abbas et al.	1265	2	[52]
6	Deep-COVID: predicting COVID-19 from chest X-ray images using deep transfer learning	Minaee et al.	1141	2	[53]
7	Using artificial intelligence to detect COVID-19 and community-acquired pneumonia based on pulmonary CT: evaluation of the diagnostic accuracy	Li et al.	1119	1	[54]
8	COVIDiagnosis-Net: deep Bayes-SqueezeNet based diagnosis of the coronavirus disease 2019 (COVID-19) from X-ray images	Ucar and Korkmaz	911	2	[55]
9	Deep learning approaches for COVID-19 detection based on chest X-ray images	Ismael and Şengür	827	6	[56]
10	COVID-CAPS: a capsule network-based framework for identification of COVID-19 cases from X-ray images	Afshar et al.	803	1	[57]
11	A combined deep CNN-LSTM network for the detection of novel coronavirus (COVID-19) using X-ray images	Islam et al.	763	6	[58]
12	Multi-task deep learning based CT imaging analysis for COVID-19 pneumonia: classification and segmentation	Amyar et al.	667	3	[59]
13	JCS: an explainable COVID-19 diagnosis system by joint classification and segmentation	Wu et al.	545	2	[60]
14	COVID-19 detection through transfer learning using multimodal imaging data	Horry et al.	526	4	[61]
15	Improving the performance of CNN to predict the likelihood of COVID-19 using chest X-ray images with preprocessing algorithms	Heidari et al.	475	4	[62]
16	Automatic detection of coronavirus disease (COVID-19) in X-ray and CT images: a machine learning based approach	Kassania et al.	442	3	[63]
17	Deep transfer learning-based automated detection of COVID-19 from lung CT scan slices	Ahuja et al.	431	4	[64]
18	Using X-ray images and deep learning for automated detection of coronavirus disease	El Asnaoui and Chawki	406	2	[65]
19	Extracting possibly representative COVID-19 biomarkers from X-ray images with deep learning approach and image data related to pulmonary diseases	Apostolopoulos et al.	381	6	[66]
20	A light CNN for detecting COVID-19 from CT scans of the chest	Polsinelli et al.	365	2	[67]

Dataset Collection

For the 450 articles covered by this bibliometric analysis, 172 different datasets were used. Some of these are collected by specific institutions, while others are combinations of datasets from various sources. Tizhoosh and Fratesi, in their study, have mentioned the fact that the availability of high-quality COVID-19 images that has been properly diagnosed is still very limited. In fact, it has been argued that many publicly available "toy" datasets do not comply with clinical standards and a well-curated dataset needs to involve professional radiologists and should be undertaken in multiple phases that span over one week's time [68]. This clearly highlights that, even within a single journal like PubMed and over a relatively short bibliometric timeframe, there is a significant lack of consistent and high-quality medical data. This deficiency makes it difficult to properly evaluate the performance of deep learning models, forcing researchers to rely on over 172 different datasets. Therefore, one of the major objectives of this bibliometric review is to allow the research community to have updates on available and reliable datasets. Table 5 shows the top 10 databases with images verified by renowned institutions and updated regularly.

**Table 5:** Publicly available COVID-19 imaging datasets C: COVID-19; P: pneumonia; N: normal; LO: lung opacity; VP: viral pneumonia; ML: mild COVID-19 pneumonia; MD: moderate COVID-19 pneumonia; SC: severe COVID-19 pneumonia; CC: critical COVID-19 pneumonia; CC-CCII: China Consortium of Chest CT Image Investigation; iCTCF: integrative resource of chest computed tomography images and clinical features; CXR: chest X-ray; CT: computed tomography

Name	Source	Type	Classes	Sample size	Link
CC-CCII	National hospitals (China)	CT	C, P, N	411,529	[69]
COVID-19 Radiography Database (KAGGLE)	Universities of Doha, Qatar, Dhaka, Bangladesh with their collaborators from Pakistan and Malaysia	CXR	C, LO, VP, N	21,165	[70]
SARS-COV-2 CT Scan	Sao Paulo hospitals (Brazil)	CT	C, N	2482	[71]
COVIDx	COVID-19 IDC (Canada), MIDRC (USA), Actualmed (Spain), COVID-19 RD, RSNA PDC (USA), COVID-19 CXR (Canada)	CXR	C, N	84,818	[72]
COVIDx-CT	ITAC (Canada), LIDC-IDRI, Radiopaedia (Australia)	CT	C, N, P	425,024	[73]
MosMedData	Municipal hospitals in Moscow (Russia)	CT	ML, MD, SC, CC, N	1000	[74]
iCTCF	China National Center for Bioinformation	CT	C, I, ME, Z, MP	5822	[75]
QaTa-COV19 Dataset	Qatar University and Tampere University and Hamad Medical Corporation	CXR	C	9258	[76]
CheXpert: Chest X-rays	Stanford Center for Artificial Intelligence in Medicine and Imaging (AIMI), USA	CXR	P	65240	[77]
BIMCV-COVID19+	Medical Imaging Databank in Valencian Region MIB (Spain)	CT, CXR	C, P	23527	[78]

Discussions

We conducted a rigorous and comprehensive bibliometric analysis of publications related to COVID-19 medical imaging, covering the period from January 1, 2020, to November 1, 2024. The keyword analysis revealed that deep learning has emerged as a prominent area of research in this field. Deep learning networks represent some of the most advanced methodologies for medical image analysis, aiming to provide reliable diagnostic support to healthcare professionals and alleviate high clinical workloads, particularly in situations where healthcare systems are overwhelmed or in resource-limited settings facing a shortage of radiologists [79].

However, the diagnostic performance of deep learning models does not demonstrate a significant advantage over that of experienced radiologists. In terms of interpretation time, deep learning algorithms require approximately 10 seconds per CT scan for analysis and reporting, whereas an experienced radiologist typically spends around 10 minutes on the same task [79].

Regarding authorship, researchers from China ranked among the top five most prolific authors based on publication output. As COVID-19 was first identified in Wuhan, China, the earliest contributions in this field predominantly originated from Chinese authors. This reflects the prompt and extensive mobilization of Chinese research institutions in investigating both the clinical manifestations and radiological characteristics of the disease at its point of origin in Wuhan [6]. These findings are consistent with those reported by Wen et al. [80], who demonstrated that China and the United States led globally in both the total number of publications and representative research institutions in COVID-19-related imaging.

In terms of journals, Elsevier ranked among the leading contributors in this field, with 153 publications, making it the most influential publisher during the bibliometric analysis period. We further analyzed the datasets used across the reviewed publications. CNN models require large-scale datasets encompassing broad data variation to achieve optimal diagnostic accuracy. However, the limited availability of such datasets remains a significant obstacle to effective model training. Consequently, most models have been developed and validated using relatively small datasets.

Notably, the majority of these datasets are no longer publicly accessible. Of the 172 datasets identified across the reviewed publications, 95 were recomposed databases (55.23%), 40 were no longer available (23.25%), and only 21.51% originated from reliable sources such as the CC-CCII and the Radiological Society of North America. For this reason, we curated these datasets and provided the research community with a regularly updated list to ensure data consistency and enable the accurate evaluation of the true performance of various deep learning models on standardized data.

This study has several limitations. First, because it focused on analyzing co-citation relationships, only the PubMed database was used, which may have restricted the scope of included literature. As a result, some relevant publications might have been omitted, potentially leading to an underestimation of citation counts. Second, the use of VOSviewer in conjunction with the PubMed database presents certain limitations, particularly in conducting a comprehensive analysis of countries, institutions, and authors, as it does not account for all co-authorship affiliations. Third, while we quantified class imbalance across datasets, we did not systematically extract or compare the imbalance mitigation strategies used in the reviewed studies.

Although numerous models have been proposed, enhanced interoperability and greater data sharing across sources could further improve performance. Looking ahead, future research should aim to strengthen the model's capacity to detect multiple pathologies simultaneously and accurately by integrating heterogeneous datasets. Furthermore, COVID-19-related imaging research is gradually shifting toward the investigation of other pulmonary diseases, leveraging emerging technologies, innovative analytical methods, and quantitative approaches to examine microstructural alterations and changes in lung tissue.

## Conclusions

Although the COVID-19 pandemic has had profound global impacts, it has served as a significant catalyst for the integration of AI in the field of medical imaging. This review has highlighted the considerable heterogeneity present within COVID-19-specific datasets. Nonetheless, current imaging research is progressively shifting focus toward analyzing the adverse effects of the virus, particularly in the evaluation, diagnosis, and management of tumor pathologies, chronic diseases, and degenerative conditions. In this context, radiologists face the significant challenge of establishing standardized guidelines or consensus protocols for the imaging-based assessment of systemic alterations associated with COVID-19 and other pathologies.

The role of AI in medical imaging goes beyond just analyzing images. Recent innovations include automated interpretations of medical images, analyzing text from electronic health records, and self-labeling and self-reporting by patients. These innovations empower healthcare professionals to gain deeper insights from patient data, resulting in more accurate diagnoses and effective treatment strategies. Collectively, these innovations highlight how AI can reshape medical imaging and healthcare delivery. However, as the field continues to evolve, addressing existing limitations and ensuring smooth integration into clinical practice will remain critical to unlocking the full potential of AI in improving patient care. In this context, one of the primary objectives of the present bibliometric analysis is to provide the scientific community with an updated overview of accessible, reliable, and research-relevant datasets.
